# Endoscopic Ultrasound-Guided Biliary Drainage

**DOI:** 10.3390/jcm12072736

**Published:** 2023-04-06

**Authors:** John B. Doyle, Amrita Sethi

**Affiliations:** Division of Digestive and Liver Diseases, Columbia University Irving Medical Center, New York-Presbyterian Hospital, New York, NY 10032, USA

**Keywords:** endoscopic ultrasound, biliary obstruction, choledochoduodenostomy, hepaticogastrostomy

## Abstract

Endoscopic retrograde cholangiopancreatography (ERCP) and percutaneous transhepatic biliary drainage (PTBD) are currently first- and second-line therapeutic options, respectively, for the relief of biliary obstruction. In recent years, however, endoscopic ultrasound-guided biliary drainage (EUS-BD) has become an established alternative therapy for biliary obstruction. There are multiple different techniques for EUS-BD, which can be distinguished based on the access point within the biliary tree (intrahepatic versus extrahepatic) and the location of stent placement (transenteric versus transpapillary). The clinical and technical success rates of biliary drainage for EUS-BD are similar to both ERCP and PTBD, and complication rates are favorable for EUS-BD relative to PTBD. As EUS-BD becomes more widely practiced and endoscopic tools continue to advance, the outcomes will likely improve, and the breadth of indications for EUS-BD will continue to expand.

## 1. Introduction

Endoscopic retrograde cholangiopancreatography (ERCP) is currently the first-line therapeutic option for the relief of benign and malignant biliary obstruction [1]. During ERCP, a side-viewing duodenoscope is used to cannulate the ampulla of Vater, through which the biliary tree and pancreatic duct can be accessed for dilation or stent placement. However, ERCP is unsuccessful in relieving biliary obstruction in 5–10% of cases [2,3]. This is often due to anatomical abnormalities or post-surgical changes that render cannulating the ampulla either difficult or impossible.

For decades, the second-line therapeutic intervention for biliary drainage following a failed ERCP has been percutaneous transhepatic biliary drainage (PTBD). In PTBD, the biliary system is accessed via a cutaneous incision, and biliary obstruction is relieved by an external biliary drain [4]. PTBD can have notable complications, including bacteremia, hemobilia, and the dislodgement, occlusion, or leakage of the external biliary drain [5,6,7]. Relative to internal enteric biliary drainage, the presence of an external biliary catheter that is required in PTBD can also lead to frequent bag exchanges, skin irritation, and reduced quality of life [8,9,10].

In recent years, endoscopic ultrasound-guided biliary drainage (EUS-BD) has been recognized as an appealing alternative to PTBD to relieve biliary obstruction after failed ERCP. In this review, we highlight the current indications, techniques, and outcomes of EUS-BD. We also discuss its potential as a primary option for biliary drainage as new endoscopic tools can improve the feasibility and accessibility of EUS-BD.

## 2. EUS-BD: Indications and Technique

EUS-BD was first described in 2001 by Giovannini et al., who reported the successful drainage of the common bile duct with a transduodenal plastic stent [11]. In the two decades since, techniques have been refined and expanded, and EUS-BD has become an essential endoscopic therapy for patients with biliary obstruction.

At present, EUS-BD is most commonly indicated for patients with malignant obstruction of the distal biliary tree when ERCP is unsuccessful or not feasible. This is often due to anatomical pathology, which makes it difficult or impossible to cannulate the papilla with a side-viewing duodenoscope, including gastric outlet obstruction, duodenal stenosis, ampullary tumor, or periampullary diverticulum. In addition, EUS-BD is useful for patients with surgically-altered anatomy, particularly following surgeries such as Roux-en-Y gastric bypass, Roux-en-Y hepaticojejunostomy, pancreaticoduodenectomy, or partial gastrectomy, in which access to the ampulla is technically cumbersome [12,13]. EUS-BD has also been used in patients with existing gastroduodenal stents that obstruct ampullary access [14].

There are multiple different techniques for EUS-BD, which have been distinguished based on their access point within the biliary tree (intrahepatic vs. extrahepatic) and the location of stent placement (transenteric vs. transpapillary). The choice of technique is based largely on patient anatomy and operator expertise [15,16].

### 2.1. EUS-Guided Hepaticogastrostomy (EUS-HGS)

In this technique, an echoendoscope is positioned in the gastric body to provide the ultrasound visualization of the left intrahepatic bile ducts [16,17,18,19]. Under this ultrasound visualization, a needle is used to access the intrahepatic biliary ducts, and a color doppler is used to identify and avoid any intervening vasculature. After needle access is obtained, a cholangiogram is performed to confirm biliary access and delineate biliary anatomy. A guidewire is then advanced through the needle and into the intrahepatic duct and biliary tree. After dilation, a stent can be deployed over the guidewire to create a hepaticogastrostomy and allow bile drainage directly into the stomach lumen.

EUS-HGS is particularly useful for patients with a gastroduodenal obstruction or post-surgical anatomy, including patients with prior pancreaticoduodenectomy or Roux-en-Y hepaticojejunostomy [16]. Given that EUS-HGS techniques typically involve access to the dilated left intrahepatic biliary ducts, the utility of EUS-HGS may be more limited in patients without intrahepatic ductal dilation or with only a right-sided intrahepatic biliary obstruction [18]. Relative contraindications include coagulopathy, massive ascites, and stomach wall pathology, such as a tumor or ulceration [16]. The most common complications of EUS-HGS include infection (including cholangitis, pancreatitis, and biliary peritonitis), bleeding, and bile leaks.

### 2.2. EUS-Guided Choledochoduodenostomy (EUS-CDS)

In EUS-CDS, an echoendoscope is positioned in the duodenal bulb, and a needle is placed into the extrahepatic biliary tree under direct ultrasound guidance. In a similar fashion to the EUS-HGS technique, a contrast is then injected to obtain a cholangiogram, and a guidewire is inserted into the common hepatic duct or the common biliary duct. A fistulous tract is created with cautery or dilation, and a stent is deployed [17,18,19]. The result is a transduodenal stent draining the extrahepatic biliary tree, as opposed to EUS-HGS, which results in a transgastric stent draining the intrahepatic biliary tree.

EUS-CDS can be a useful technique for biliary drainage in patients with distal malignant biliary obstruction due to periampullary malignancy and mass or papillary stenosis. It has similar complications and contraindications to EUS-HGS. If performed in the setting of a pending or existing duodenal obstruction, then adequate bile drainage needs to be established, either with a duodenal stent or a gastrojejunostomy, which can be performed endoscopically at the time of EUS-CDS. In recent years, lumen-apposing metal stents (LAMS) have been increasingly used in EUS-CDS, which can improve anastomotic creation and anchoring between the enteric lumen and the biliary tree [12,20,21]. Electrocautery-enhanced LAMS, in particular, allows for a single-stage biliary puncture and stent placement and, thus, has the potential to decrease the procedure difficulty and complication risk [21,22].

### 2.3. EUS-Guided Antegrade Stent Placement

While EUS-HGS and EUS-CDS both involve transenteric stenting and biliary drainage, EUS-guided antegrade stent placement is a technique that can achieve transpapillary biliary stenting. In this technique, either intrahepatic or extrahepatic access is created via the gastric or duodenal lumen under ultrasound guidance, as described above. Once the biliary tree is accessed, a guidewire can be used to traverse the biliary obstruction and the ampulla [16,17]. Contrast can be injected to confirm extravasation into the small bowel to ensure proper placement, and if confirmed, a transpapillary stent can be placed in the antegrade fashion. This technique requires that a guidewire is able to pass distally to the obstructed biliary tree.

EUS-guided antegrade stent placement has a theoretical advantage over EUS-HGS or EUS-CDS in that it can avoid the creation of a new anastomosis at the biliary access site and any consequent adverse events [12]. EUS-guided antegrade stent placement can be especially useful in patients with surgically-altered anatomies, such as the Roux-en-Y gastric bypass, and who have preserved ampullary anatomy and physiology. With a transpapillary stent, however, there is a higher risk of pancreatitis or cholangitis that is relative to EUS-HGS or EUS-CG [12]. In patients with suitable anatomy, EUS-guided antegrade stent placement can also be combined with EUS-HGS; relative to EUS-HGS alone, this combined technique has the potential advantages of decreased adverse events (such as bile peritonitis) and prolonged stent patency [23].

### 2.4. EUS-Guided Rendezvous Technique

In the EUS-guided rendezvous technique, extrahepatic or intrahepatic access can be obtained using the echoendoscope and the methods described above. Similar to EUS-guided antegrade stent placement, once biliary access is obtained and a guidewire is placed across the biliary obstructions, across the ampulla, and into the small bowell. The guidewire is then left in place, and a duodenoscope is maneuvered to the second portion of the duodenum; the wire is used to facilitate ampullary cannulation, and a conventional ERCP can then be performed.

As with the EUS-guided antegrade stent placement, this achieves transpapillary drainage without transluminal anastomosis at the biliary access site [12,18,19]. This rendezvous technique can be useful when the second portion of the duodenum is accessible, but the conventional cannulation of the papilla is technically difficult [12,17,19].

## 3. Outcomes of EUS-BD

### 3.1. Efficacy and Adverse Events of EUS-BD

EUS-BD has a high technical and clinical success rate in relieving biliary obstruction, along with a favorable adverse event rate profile that is relative to alternative interventions. Much of the current literature has explored the role of EUS-BD after failed ERCP in the relief of MBO in particular. Systematic reviews and meta-analyses have demonstrated the technical and clinical success rates of EUS-BD to be 90–95% in this setting, respectively [8,18,24]. Meta-analyses have demonstrated procedure-related adverse event rates to be between 15 and 24%, with the most common complications being infection (including cholangitis, pancreatitis, and biliary peritonitis), bleeding, pneumoperitoneum, and bile leaks [8,18,24,25]. In EUS-HGS, a transesophageal puncture has also been reported, which can result in pneumothorax or mediastinitis [25].

The optimal technique for EUS-BD remains unclear, as it is difficult to compare different biliary access sites or the direction of stent placement, given the heterogeneity of patient populations and the relative rarity of each technique. Two randomized controlled trials (n = 49 and 47, respectively) have compared EUS-HGS to EUS-CDS for distal MBO after failed ERCP, and neither found significant differences in terms of technical or clinical success, adverse event rates, or morbidity [26,27]. One recent multicenter retrospective review (n = 182) found that choledochoduodenostomy was associated with longer stent patency than hepaticogastrostomy but otherwise noted a similar efficacy between the two approaches [28]. Other retrospective reviews and meta-analyses have demonstrated similar or conflicting results [12,24,29]. An ongoing randomized, multicenter clinical comparison between EUS-HGS and EUS-guided antegrade stent placement may shed light on the differences between these approaches [30]. Currently, however, the literature comparing different EUS-BD techniques is limited, so the optimal approach at many centers remains dependent upon patient anatomy and endoscopist expertise.

### 3.2. EUS-BD vs. PTBD

Given that PTBD remains the conventional therapeutic intervention for biliary obstruction following failed ERCP, investigators have compared the outcomes between PTBD and EUS-BD [8,9,31,32,33,34]. Recent randomized controlled trials have found that EUS-BD and PTBD were equivalent in terms of the technical and clinical success of relieving biliary obstruction [31,34], and multiple retrospective studies and meta-analyses have demonstrated similar findings [8,9,32,33,35]. In one large meta-analysis, Moole et al. found the pooled odds ratio for successful biliary drainage in EUS-BD vs. PTBD to be 3.1 (95% CI 1.1–8.4), suggesting that EUS-BD may be even more efficacious than PTBD in patients with malignant biliary strictures [8].

The current literature also suggests that EUS-BD is associated with lower adverse events and complications than PTBD. One randomized trial found that the procedure-related adverse event rate for EUS-BD (8.8%) was significantly lower than that for PTBD (31.2%) [31]; a second found a lower rate of re-intervention for EUS-BD [34]. Retrospective studies and meta-analyses have found similar results, with EUS-BD demonstrating lower infectious complications [8], fewer repeat interventions [9,35], and less post-procedural pain [35]. Other postulated advantages of EUS-BD over PTBD include improved patient quality of life (given the lack of an external catheter) and the ability to perform EUS-BD in the same session as a failed ERCP [8,10]. A multicenter, randomized trial comparison between EUS-BD and PTBD after failed ERCP for distal MBO is underway to more definitively answer these questions, which will be the largest prospective trial to date [36].

### 3.3. EUS-BD vs. ERCP as First-Line Intervention for Malignant Biliary Obstruction

Although EUS-BD is currently considered a second-line therapy after failed ERCP, several studies in recent years have compared EUS-BD to ERCP as the first-line intervention for biliary obstruction. The theoretical advantages of transenteric stenting (via EUS-HGS or EUS-CDS) relative to transpapillary stenting via ERCP include: the minimization of papillary manipulation leading to pancreatitis; the avoidance of stent tumor ingrowth which can occur when the stent is placed through a distal malignant biliary stricture; and the ability to access biliary ducts despite surgically-altered anatomy or gastroduodenal stents.

Meta-analyses have found that EUS-BD and ERCP have similarly high rates of technical success and clinical success when used as the primary option for biliary obstruction [25]. EUS-BD and ERCP also have similar rates of adverse events; while bile peritonitis remains a concern in EUS-BD (occurring in up to 2.4% of cases), EUS-BD has significantly lower rates of post-procedure pancreatitis and stent patency relative to ERCP. This was demonstrated in a randomized, controlled multicenter trial (n = 125) which found that EUS-BD was non-inferior to ERCP as a primary option for MBO; the study also found lower rates of overall adverse events for EUS-BD relative to ERCP (6.3% vs. 19.7%, respectively), including post-procedure pancreatitis (0 vs. 14.8%) and reintervention (15.6% vs. 42.6%), as well as a higher rate of stent patency (85.1% vs. 48.9%) with EUS-BD [37]. EUS-BD (specifically EUS-HGS and EUS-CG) may also have superior technical success to ERCP in patients with indwelling gastroduodenal stents who develop a subsequent biliary obstruction [14].

## 4. Limitations and Future Directions in EUS-BD

A major barrier to the widespread adoption of EUS-BD is operator expertise relative to PTBD, which is more widely practiced in most parts of the world [18,25]. Relative to ERCP or PTBD, EUS-BD is a relatively new therapeutic intervention that is practiced mainly at tertiary care centers. As such, EUS-BD is associated with a notable learning curve for endoscopists. For instance, in one cohort of 101 patients undergoing EUS-BD in a single center between 2006 and 2013, there were six procedure-related deaths; five of these deaths were among the first 50 patients in which the procedure was performed, and only one death was among the last 51 patients [29]. This may be true on a population level as well: one meta-analysis on different EUS-BD approaches found that studies published after 2013 had a higher technical success rate than those published prior to 2013 [24]. It seems likely that EUS-BD outcomes will continue to improve as endoscopist experience increases and adoption expands.

New endoscopic devices are likely to improve operability and clinical success rates for EUS-BD. While early studies have lacked the tools specific to EUS-BD and have instead relied on devices borrowed from other procedures, new endoscopic stents, and dilators have already changed how EUS-BD is performed. EUS-BD originally relied on traditional plastic stents; for instance, newly designed plastic stents with a tapered tip and four flanges with pigtail anchors have been developed specifically for EUS-HGS and have demonstrated good technical and clinical success [38]. Similarly, the adoption of newer covered self-expanding metal stents (CSEMS) has been associated with significantly lower adverse events in EUS-BD over time [24]. As noted previously, electrocautery-enhanced LAMS delivery systems have been increasingly used in EUS-CDS, which allow for single-step biliary access and stent placement with high technical success rates and acceptable adverse event rates [12,20,21]. Data from a recent large nationwide analysis of EUS-CDS with LAMS demonstrated reproducible efficacy and safety across different centers with a range of endoscopist expertise, suggesting that technological advancements such as LAMS have the potential to democratize the utilization of EUS-BD techniques beyond tertiary medical centers [21]. Other technological advancements—such as stent anti-migratory systems [39] and drill dilators, which are specific for intrahepatic bile ducts [40]—are expected to continue to shape the way EUS-BD is performed.

Ultimately, as endoscopic tools for EUS-BD continue to advance and EUS-BD becomes more widely practiced, the utilization and indications for EUS-BD are likely to expand. As noted above, research trials are already underway to determine which patients would benefit from EUS-BD rather than ERCP as the first-line option for biliary obstruction. Other areas that are being explored include using EUS-BD as a preferred method for gallbladder drainage in patients who are not surgical candidates [41,42,43]. EUS-BD also has the potential to become the preferred pre-operative management for MBO in patients ultimately undergoing surgery [44]. Taken together, EUS-BD and related techniques have the potential to transform the current paradigms that define how patients with hepatobiliary diseases are treated. 

## Data Availability

Not applicable.

## References

[1] Early D.S., Ben-Menachem T., Decker G.A., Evans J.A., Fanelli R.D., Fisher D.A., Fukami N., Hwang J.H., Jain R., Jue T.L. (2012). Appropriate use of GI endoscopy. Gastrointest. Endosc..

[2] Enochsson L., Swahn F., Arnelo U., Nilsson M., Löhr M., Persson G. (2010). Nationwide, population-based data from 11,074 ERCP procedures from the Swedish Registry for Gallstone Surgery and ERCP. Gastrointest. Endosc..

[3] Dumonceau J.M., Tringali A., Papanikolaou I.S., Blero D., Mangiavillano B., Schmidt A., Vanbiervliet G., Costamagna G., Devière J., García-Cano J. (2018). Endoscopic biliary stenting: Indications, choice of stents, and results: European Society of Gastrointestinal Endoscopy (ESGE) Clinical Guideline–Updated October 2017. Endoscopy.

[4] Kavanagh P.V., van Sonnenberg E., Wittich G.R., Goodacre B.W., Walser E.M. (1997). Interventional Radiology of the Biliary Tract. Endoscopy.

[5] Oh H.-C., Lee S., Lee T., Kwon S., Lee S.S., Seo D.-W., Kim M.-H. (2007). Analysis of percutaneous transhepatic cholangioscopy-related complications and the risk factors for those complications. Endoscopy.

[6] Ginat D., Saad W.E.A., Davies M.G., Saad N.E., Waldman D.L., Kitanosono T. (2011). Incidence of Cholangitis and Sepsis Associated With Percutaneous Transhepatic Biliary Drain Cholangiography and Exchange: A Comparison Between Liver Transplant and Native Liver Patients. AJR Am. J. Roentgenol..

[7] Molina H., Chan M.M., Lewandowski R.J., Gabr A., Riaz A. (2021). Complications of Percutaneous Biliary Procedures. Semin. Interv. Radiol..

[8] Moole H., Bechtold M.L., Forcione D., Puli S.R. (2017). A meta-analysis and systematic review: Success of endoscopic ultrasound guided biliary stenting in patients with inoperable malignant biliary strictures and a failed ERCP. Medicine.

[9] Khashab M.A., El Zein M.H., Sharzehi K., Marson F.P., Haluszka O., Small A.J., Nakai Y., Park D.H., Kunda R., Teoh A.Y. (2016). EUS-guided biliary drainage or enteroscopy-assisted ERCP in patients with surgical anatomy and biliary obstruction: An international comparative study. Endosc. Int. Open.

[10] Park D.H., Nam K., Kim D.U., Lee T.H., Iwashita T., Nakai Y., Bolkhir A., Castro L.A., Vazquez-Sequeiros E., De La Serna C. (2018). Patient perception and preference of EUS-guided drainage over percutaneous drainage when endoscopic transpapillary biliary drainage fails: An international multicenter survey. Endosc. Ultrasound.

[11] Giovannini M., Moutardier V., Pesenti C., Bories E., Lelong B., Delpero J.R. (2001). Endoscopic Ultrasound-Guided Bilioduodenal Anastomosis: A New Technique for Biliary Drainage. Endoscopy.

[12] Canakis A., Baron T.H. (2020). Relief of biliary obstruction: Choosing between endoscopic ultrasound and endoscopic retrograde cholangiopancreatography. BMJ Open Gastroenterol..

[13] Elfert K., Zeid E., Duarte-Chavez R., Kahaleh M. (2022). Endoscopic ultrasound guided access procedures following surgery. Best Pr. Res. Clin. Gastroenterol..

[14] Yamao K., Kitano M., Takenaka M., Minaga K., Sakurai T., Watanabe T., Kayahara T., Yoshikawa T., Yamashita Y., Asada M. (2018). Outcomes of endoscopic biliary drainage in pancreatic cancer patients with an indwelling gastroduodenal stent: A multicenter cohort study in West Japan. Gastrointest. Endosc..

[15] Tyberg A., Desai A.P., Kumta N.A., Brown E., Gaidhane M., Sharaiha R.Z., Kahaleh M. (2016). EUS-guided biliary drainage after failed ERCP: A novel algorithm individualized based on patient anatomy. Gastrointest. Endosc..

[16] Boulay B.R., Lo S.K. (2018). Endoscopic Ultrasound–Guided Biliary Drainage. Gastrointest. Endosc. Clin. N. Am..

[17] Mishra A., Tyberg A. (2019). Endoscopic ultrasound guided biliary drainage: A comprehensive review. Transl. Gastroenterol. Hepatol..

[18] Nussbaum J.S., Kumta N.A. (2019). Endoscopic Ultrasound-Guided Biliary Drainage. Gastrointest. Endosc. Clin. N. Am..

[19] Dietrich C., Braden B., Burmeister S., Aabakken L., Arciadacono P., Bhutani M., Götzberger M., Healey A., Hocke M., Hollerbach S. (2022). How to perform EUS-guided biliary drainage. Endosc. Ultrasound.

[20] Krishnamoorthi R., Dasari C.S., Chandrasekar V.T., Priyan H., Jayaraj M., Law J., Larsen M., Kozarek R., Ross A., Irani S. (2020). Effectiveness and safety of EUS-guided choledochoduodenostomy using lumen-apposing metal stents (LAMS): A systematic review and meta-analysis. Surg. Endosc..

[21] Fugazza A., Fabbri C., Di Mitri R., Petrone M.C., Colombo M., Cugia L., Amato A., Forti E., Binda C., Maida M. (2022). EUS-guided choledochoduodenostomy for malignant distal biliary obstruction after failed ERCP: A retrospective nationwide analysis. Gastrointest. Endosc..

[22] Yi H., Liu Q., He S., Zhong L., Wu S.-H., Guo X.-D., Ning B. (2022). Current uses of electro-cautery lumen apposing metal stents in endoscopic ultrasound guided interventions. Front. Med..

[23] Ogura T., Kitano M., Takenaka M., Okuda A., Minaga K., Yamao K., Yamashita Y., Hatamaru K., Noguchi C., Gotoh Y. (2018). Multicenter prospective evaluation study of endoscopic ultrasound-guided hepaticogastrostomy combined with antegrade stenting (with video). Dig. Endosc..

[24] Wang K., Zhu J., Xing L., Wang Y., Jin Z., Li Z. (2016). Assessment of efficacy and safety of EUS-guided biliary drainage: A systematic review. Gastrointest. Endosc..

[25] Jin Z., Wei Y., Lin H., Yang J., Jin H., Shen S., Zhang X. (2020). Endoscopic ultrasound-guided versus endoscopic retrograde cholangiopancreatography-guided biliary drainage for primary treatment of distal malignant biliary obstruction: A systematic review and meta-analysis. Dig. Endosc..

[26] Artifon E.L., Marson F.P., Gaidhane M., Kahaleh M., Otoch J.P. (2015). Hepaticogastrostomy or choledochoduodenostomy for distal malignant biliary obstruction after failed ERCP: Is there any difference?. Gastrointest. Endosc..

[27] Minaga K., Ogura T., Shiomi H., Imai H., Hoki N., Takenaka M., Nishikiori H., Yamashita Y., Hisa T., Kato H. (2019). Comparison of the efficacy and safety of endoscopic ultrasound-guided choledochoduodenostomy and hepaticogastrostomy for malignant distal biliary obstruction: Multicenter, randomized, clinical trial. Dig. Endosc..

[28] Kahaleh M., Tyberg A., Napoleon B., Robles-Medranda C., Shah J., Bories E., Kumta N., Yague A., Vazquez-Sequeiros E., Lakhtakia S. (2022). Hepaticogastrostomy versus choledochoduodenostomy: An international multicenter study on their long-term patency. Endosc. Ultrasound.

[29] Poincloux L., Rouquette O., Buc E., Privat J., Pezet D., Dapoigny M., Bommelaer G., Abergel A. (2015). Endoscopic ultrasound-guided biliary drainage after failed ERCP: Cumulative experience of 101 procedures at a single center. Endoscopy.

[30] Liao Y., Giovannini M., Zhong N., Xiao T., Sheng S., Wu Y., Zhang J., Wang S., Liu X., Sun S. (2020). Comparison of endoscopic ultrasound-guided hepaticogastrostomy and the antegrade technique in the management of unresectable malignant biliary obstruction: Study protocol for a prospective, multicentre, randomised controlled trial. Trials.

[31] Lee T.H., Choi J.-H., Park D.H., Song T.J., Kim D.U., Paik W.H., Hwangbo Y., Lee S.S., Seo D.W., Lee S.K. (2016). Similar Efficacies of Endoscopic Ultrasound–guided Transmural and Percutaneous Drainage for Malignant Distal Biliary Obstruction. Clin. Gastroenterol. Hepatol..

[32] Sharaiha R.Z., Khan M.A., Kamal F., Tyberg A., Tombazzi C.R., Ali B., Tombazzi C., Kahaleh M. (2017). Efficacy and safety of EUS-guided biliary drainage in comparison with percutaneous biliary drainage when ERCP fails: A systematic review and meta-analysis. Gastrointest. Endosc..

[33] Baniya R., Upadhaya S., Madala S., Subedi S.C., Mohammed T.S., Bachuwa G. (2017). Endoscopic ultrasound-guided biliary drainage versus percutaneous transhepatic biliary drainage after failed endoscopic retrograde cholangiopancreatography: A meta-analysis. Clin. Exp. Gastroenterol..

[34] Marx M., Caillol F., Autret A., Ratone J.-P., Zemmour C., Boher J.M., Pesenti C., Bories E., Barthet M., Napoléon B. (2022). EUS-guided hepaticogastrostomy in patients with obstructive jaundice after failed or impossible endoscopic retrograde drainage: A multicenter, randomized phase II Study. Endosc. Ultrasound.

[35] Hassan Z., Gadour E. (2022). Systematic review of endoscopic ultrasound-guided biliary drainage versus percutaneous transhepatic biliary drainage. Clin. Med..

[36] Schmitz D., Valiente C.T., Dollhopf M., Perez-Miranda M., Küllmer A., Gornals J., Vila J., Weigt J., Voigtländer T., Redondo-Cerezo E. (2022). Percutaneous transhepatic or endoscopic ultrasound-guided biliary drainage in malignant distal bile duct obstruction using a self-expanding metal stent: Study protocol for a prospective European multicenter trial (PUMa trial). PLoS ONE.

[37] Paik W.H., Lee T.H., Park D.H., Choi J.-H., Kim S.-O., Jang S., Kim D.U., Shim J.H., Song T.J., Lee S.S. (2018). EUS-Guided Biliary Drainage Versus ERCP for the Primary Palliation of Malignant Biliary Obstruction: A Multicenter Randomized Clinical Trial. Am. J. Gastroenterol..

[38] Umeda J., Itoi T., Tsuchiya T., Sofuni A., Itokawa F., Ishii K., Tsuji S., Ikeuchi N., Kamada K., Tanaka R. (2015). A newly designed plastic stent for EUS-guided hepaticogastrostomy: A prospective preliminary feasibility study (with videos). Gastrointest. Endosc..

[39] Anderloni A., Fugazza A., Spadaccini M., Colombo M., Capogreco A., Carrara S., Maselli R., Ferrara E., Galtieri P., Pellegatta G. (2023). Feasibility and safety of a new dedicated biliary stent for EUS-guided hepaticogastrostomy: The FIT study (with video). Endosc. Ultrasound.

[40] Ogura T., Uba Y., Yamamura M., Kawai J., Nishikawa H. (2023). Successful endoscopic ultrasound-guided hepaticogastrostomy with use of a novel drill dilator for challenging tract dilation. Endoscopy.

[41] Adler D., Kamal F., Khan M., Lee-Smith W., Sharma S., Acharya A., Farooq U., Aziz M., Kouanda A., Dai S.-C. (2022). Efficacy and safety of EUS-guided gallbladder drainage for rescue treatment of malignant biliary obstruction: A systematic review and meta-analysis. Endosc. Ultrasound.

[42] Robles-Medranda C., Oleas R., Puga-Tejada M., Alcivar-Vasquez J., Del Valle R., Olmos J., Arevalo-Mora M., Egas-Izquierdo M., Tabacelia D., Baquerizo-Burgos J. (2023). Prophylactic EUS-guided gallbladder drainage prevents acute cholecystitis in patients with malignant biliary obstruction and cystic duct orifice involvement: A randomized trial (with video). Gastrointest. Endosc..

[43] Hemerly M.C., de Moura D.T.H., Junior E.S.D.M., Proença I.M., Ribeiro I.B., Yvamoto E.Y., Ribas P.H.B.V., Sánchez-Luna S.A., Bernardo W.M., de Moura E.G.H. (2022). Endoscopic ultrasound (EUS)-guided cholecystostomy versus percutaneous cholecystostomy (PTC) in the management of acute cholecystitis in patients unfit for surgery: A systematic review and meta-analysis. Surg. Endosc..

[44] Mukai S., Itoi T., Tsuchiya T., Ishii K., Tonozuka R., Nagakawa Y., Kozono S., Takishita C., Osakabe H., Sofuni A. (2022). Clinical feasibility of endoscopic ultrasound-guided biliary drainage for preoperative management of malignant biliary obstruction (with videos). J. Hepato-Biliary-Pancreatic Sci..

